# Crossmodal benefits to vocal emotion perception in cochlear implant users

**DOI:** 10.1016/j.isci.2022.105711

**Published:** 2022-12-02

**Authors:** Celina Isabelle von Eiff, Sascha Frühholz, Daniela Korth, Orlando Guntinas-Lichius, Stefan Robert Schweinberger

**Affiliations:** 1 Department for General Psychology and Cognitive Neuroscience, Institute of Psychology, Friedrich Schiller University Jena, 07743 Jena, Germany; 2 Voice Research Unit, Institute of Psychology, Friedrich Schiller University Jena, 07743 Jena, Germany; 3 DFG SPP 2392 Visual Communication (ViCom), Frankfurt am Main, Germany; 4 Department of Psychology (Cognitive and Affective Neuroscience), Faculty of Arts and Social Sciences, University of Zurich, 8050 Zurich, Switzerland; 5 Department of Psychology, University of Oslo, 0373 Oslo, Norway; 6 Department of Otorhinolaryngology, Jena University Hospital, 07747 Jena, Germany

**Keywords:** Biological sciences, Neuroscience, Sensory neuroscience

## Abstract

Speech comprehension counts as a benchmark outcome of cochlear implants (CIs)—disregarding the communicative importance of efficient integration of audiovisual (AV) socio-emotional information. We investigated effects of time-synchronized facial information on vocal emotion recognition (VER). In Experiment 1, 26 CI users and normal-hearing (NH) individuals classified emotions for auditory-only, AV congruent, or AV incongruent utterances. In Experiment 2, we compared crossmodal effects between groups with adaptive testing, calibrating auditory difficulty via voice morphs from emotional caricatures to anti-caricatures. CI users performed lower than NH individuals, and VER was correlated with life quality. Importantly, they showed larger benefits to VER with congruent facial emotional information even at equal auditory-only performance levels, suggesting that their larger crossmodal benefits result from deafness-related compensation rather than degraded acoustic representations. Crucially, vocal caricatures enhanced CI users’ VER. Findings advocate AV stimuli during CI rehabilitation and suggest perspectives of caricaturing for both perceptual trainings and sound processor technology.

## Highlights

• Synchronized facial information benefits CI users' vocal emotion recognition (VER)
• This benefit seems to be due to compensatory plasticity rather than degraded hearing
• Emotional voice caricatures benefit vocal emotion recognition with a CI
• We highlight perspectives for rehabilitation and CI sound processing technology

## Graphical abstract

**Figure:**
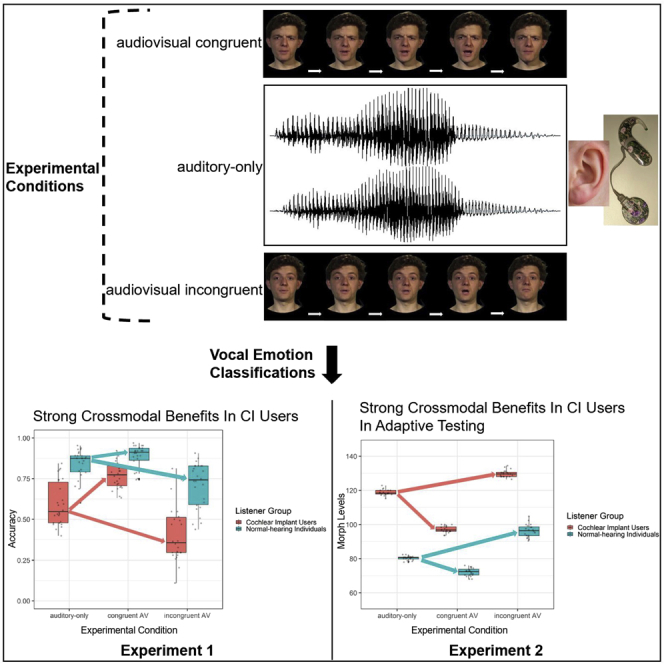

## Introduction

Many objects (e.g., people, animals, cars, or telephones) can be recognized via both auditory and visual information^1^ which may explain the important role of multisensory integration.^2^ However, audiovisual (AV) integration is particularly relevant in face-to-face social communication: Humans produce tightly corresponding facial and vocal signals that represent multisensory stimuli to which the human brain is well adapted, probably as a consequence of their frequency and daily social relevance.^3^^,^^4^ While AV integration is already considered to be ubiquitous for the perception of speech or speaker identity, many researchers take emotion perception to be especially multimodal in nature^5^^,^^6^^,^^7^ and to be particularly important to interaction.^8^ AV integration in emotion perception tends to be fast and automatic, as indicated by neurophysiological recordings which suggest early AV integration of emotional stimuli.^9^^,^^10^^,^^11^^,^^12^^,^^13^^,^^14^ Moreover, perceivers cannot well inhibit the processing of emotional information even when presented in a task-irrelevant modality.^15^^,^^16^^,^^17^

While the human brain supports remarkably efficient AV integration of spatiotemporally corresponding stimuli, the influence of sensory loss and its partial restoration on AV integration (and on crossmodal processing more generally) remains insufficiently understood. Here we investigate vocal emotion recognition (VER) with and without visual information in participants with a cochlear implant (CI)—a sensory prosthesis to treat severe hearing loss by direct electrical stimulation of the auditory nerve. Although most previous research with CI users has focused on auditory speech perception as a benchmark for implant success, recent research increasingly points to the importance of emotional communication skills in CI users.^18^ In particular, quality of life with a CI is tightly related to the ability to perceive vocal emotions.^19^^,^^20^ However, it has been controversial whether exposure to AV speech is adaptive or, in fact, maladaptive in CI users. An influential idea has been that, as a result of cortical reorganization during sensory deprivation, visual speech engages auditory cortex areas, thereby potentially interfering with auditory rehabilitation.^21^ By contrast, more recent research suggests that visual speech can provide adaptive benefits to auditory recovery with a CI.^22^ Accordingly, experts have begun to suggest that rehabilitation guidelines should encourage, rather than discourage, training with AV stimuli.^23^

More efficient processing of visual facial speech after cochlear implantation was observed by Rouger and coworkers^24^ who assumed that crossmodal plasticity in CI users allowed for efficient AV integration. According to related longitudinal data, CI users maintained higher visual-only speechreading skills than normal-hearing (NH) individuals, even years after implantation. Nevertheless, efficient AV performance in these CI users was attributed to a genuine benefit in multisensory integration.^25^ Moreover, recent research proposes that crossmodal plasticity by visual speech provides adaptive benefits to the restoration of hearing via AV processing mechanisms which potentially guide attention to auditory representations.^22^ Importantly, for present purposes, preliminary evidence suggests that crossmodal facial information could also affect CI users' processing of para- and extralinguistic social-communicative signals. For instance, CI users were strongly influenced by vision when performing auditory-gender categorizations, despite good auditory recovery.^26^ Note that these findings regarding gender perception are consistent with similar biases of CI users toward visual-predominant bimodal integration in speech perception.^27^^,^^28^^,^^29^ By contrast, results from studies investigating multimodal emotion perception in CI users are less conclusive: whereas one study^30^ found that children and adolescents with CIs did not recognize emotions better for AV stimuli than for visual-only stimuli (although NH children and adolescents did), follow-up work by the same group^31^ did identify such a perceptual benefit in children with CIs for AV stimuli, when compared to both visual-only and auditory-only stimuli. Fengler and colleagues^32^ observed that CI users with an onset of deafness before age 3 benefited more from congruent facial information than NH controls when recognizing vocal emotions. Congenitally deaf CI users showed a similar trend, but late deaf CI users did not exhibit AV benefits. For visual-only facial information, recognition rates of the CI users and their controls did not differ. Moreover, CI users experienced more costs from simultaneously presented incongruent emotional facial information than NH controls during VER.^32^

Research into VER with a CI is challenging because there is typically enormous performance variability between CI users, with some approximating the level of NH individuals and others responding close to guessing levels^18^^,^^33^^,^^34^; for a review, see Jiam et al.^18^ In the present two experiments, we studied vocal and AV emotion recognition in two groups of approximately 25 adult CI users who reflected this variability. Experiment 1 served as an initial investigation to test the hypothesis that CI users' VER can benefit from congruent facial information and that this benefit can exceed that seen in NH listeners. In addition, we sought to replicate a positive relationship between VER skills and quality of life with a CI that had been identified by recent research.^19^^,^^20^

In Experiment 2, we considered in more detail the possibility that AV benefits can depend on the baseline level of unimodal auditory performance. It therefore seemed essential to equate auditory-only performance levels between groups, even when considering findings that AV benefits to speech perception actually can be more pronounced when auditory input is intermediate, compared to when auditory input is the weakest.^35^ To achieve this, we implemented an adaptive testing procedure to individually calibrate task difficulty toward a constant performance level. This should allow a fair comparison of CI users' and NH individuals' AV integration at similar levels of auditory performance (Experiment 2). We used state-of-the-art voice morphing^36^^,^^37^ to avoid disadvantages from previous approaches to equate auditory performance. These tend to add distortion and noise or to use vocoded stimuli to simulate hearing with a CI for calibrating performance levels (e.g., Barone et al.^26^). In effect, all the present voice stimuli sound undegraded in acoustic appearance but, crucially, contain systematically different levels of diagnostic information for the critical emotion task (here, a two-alternative forced choice [2-AFC] discrimination between anger and surprise). Note that our approach is perfectly in line with recent findings that morph-based caricaturing and anti-caricaturing of vocal emotions can cause linear effects to efficiency in VER tasks across a wide range of morph levels (MLs).^38^ Experiment 2 also establishes investigations into the degree to which emotion perception with a CI can be potentially enhanced by vocal caricatures.

## Results

We performed mixed analyses of variance (ANOVAs) with repeated measures on experimental factors and a between-subject factor listener group. Note that we also considered listener sex in the initial models, but because there were no significant findings involving listener sex, we collapsed analyses across this factor (cf. STAR Methods).

### Experiment 1

#### CI users are impaired in recognizing vocal emotions, with or without AV information

**Figure 1:**
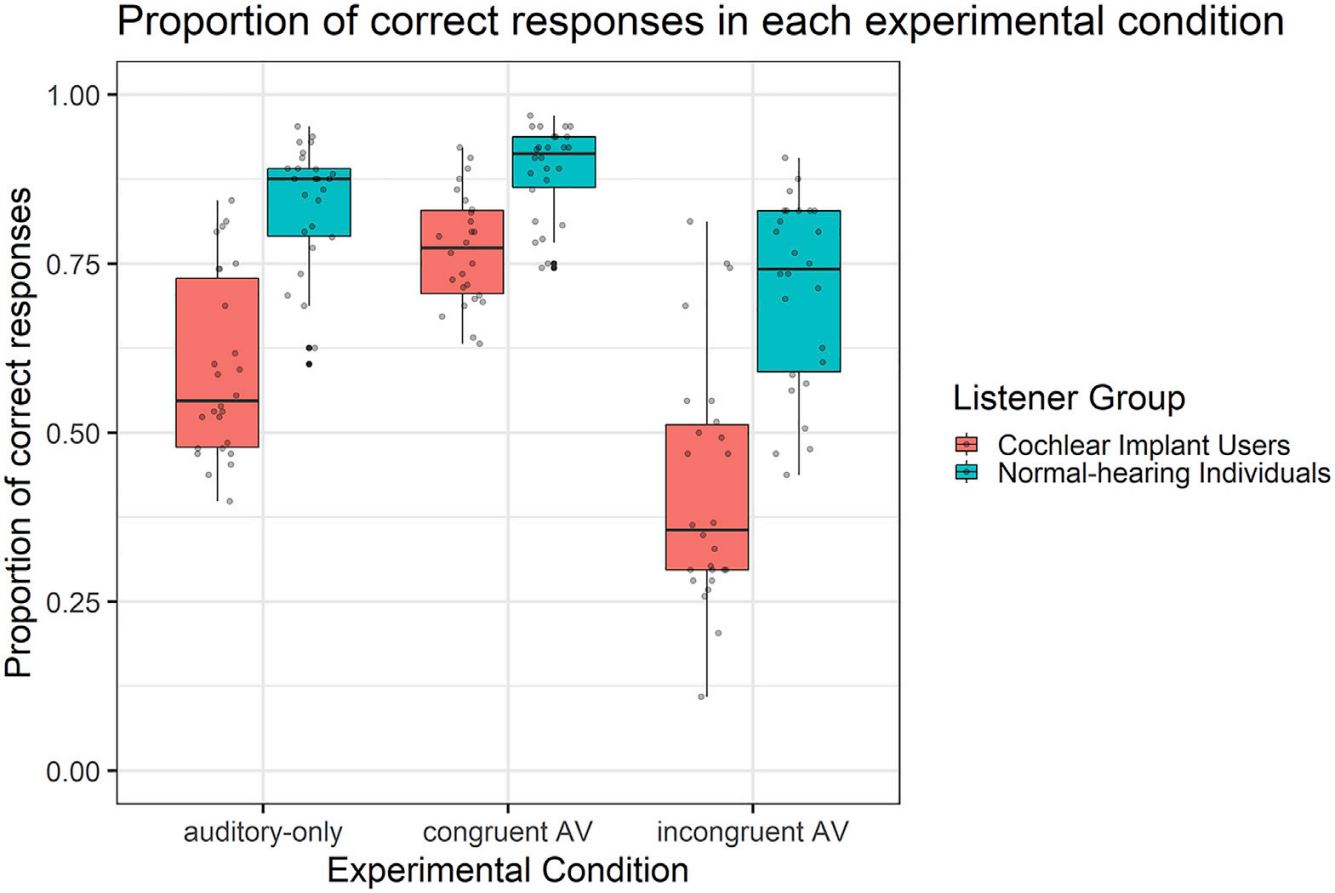
Experiment 1. Proportion of correct responses in each experimental condition (auditory-only, congruent audiovisual (AV), and incongruent audiovisual (AV)) for CI users (in red) and NH individuals (in turquoise) Note: Dots represent individual participants, lower and upper hinges correspond to the 25^th^ and 75^th^ percentiles (i.e., boxes represent the inter-quartile ranges (IQRs)), horizontal lines inside the boxes represent medians, and the upper (or lower) whiskers indicate to the largest (smallest) value no further than 1.5 ∗ IQR from the respective hinge.

CI users were substantially impaired in recognizing vocal emotions when compared to NH individuals (main effect LGroup: *F*(1, 50) = 60.485, p < 0.001, η_*p*_^*2*^ = 0.547). Figure 1 shows that this held across all presentation conditions but also suggests that the respective benefits and costs from visually congruent and incongruent facial emotions both were larger in CI users (two-way interaction LGroup x Condition, *F*(2, 100) = 13.825, p < 0.001, ε_*HF*_ = 0.707, η_*p*_^*2*^ = 0.217). Nevertheless, CI users performed significantly lower than NH individuals at each condition, including congruent AV; |*t*s(50)| ≥ 5.603, *p*s < 0.001.

#### CI users exhibit strong benefits to vocal emotion perception if congruent facial information is available

To quantify benefits and costs from AV congruent and incongruent facial information, respectively, we calculated differences between performance accuracies for congruent AV minus auditory-only trials and for incongruent AV minus auditory-only trials (for each stimulus and participant). A significant two-way interaction LGroup x Difference, *F*(1, 50) = 15.536, p < 0.001, η_*p*_^*2*^ = 0.237, indicated differences in the benefit-cost patterns of both groups. Specifically, CI users benefitted significantly more from congruent faces than NH individuals; *t*(32.031) = 4.580, p < 0.001, *Welch test*. CI users exhibited only marginally larger costs from incongruent faces; *t*(50) = −1.735, p = 0.089.

#### CI users’ ability to perceive vocal emotions is positively correlated with quality of life domain environmental health

CI users’ overall performance (i.e., VER accuracy in all conditions taken together), as well as VER accuracy in the auditory-only condition, were positively correlated with the environmental health domain of the WHOQOL-BREF (related to financial resources, safety, health, social services, living physical environment, opportunities to acquire new skills and knowledge, recreation, general environment, and transportation), with *r*_*s*_ = 0.41, p = 0.043, n = 25 and *r*_*s*_ = 0.41, p = 0.040, n = 25, respectively. For full details, see supplemental materials, 5.1.3.

### Experiment 2

#### CI users are impaired in vocal emotion recognition, with or without AV information

**Figure 2:**
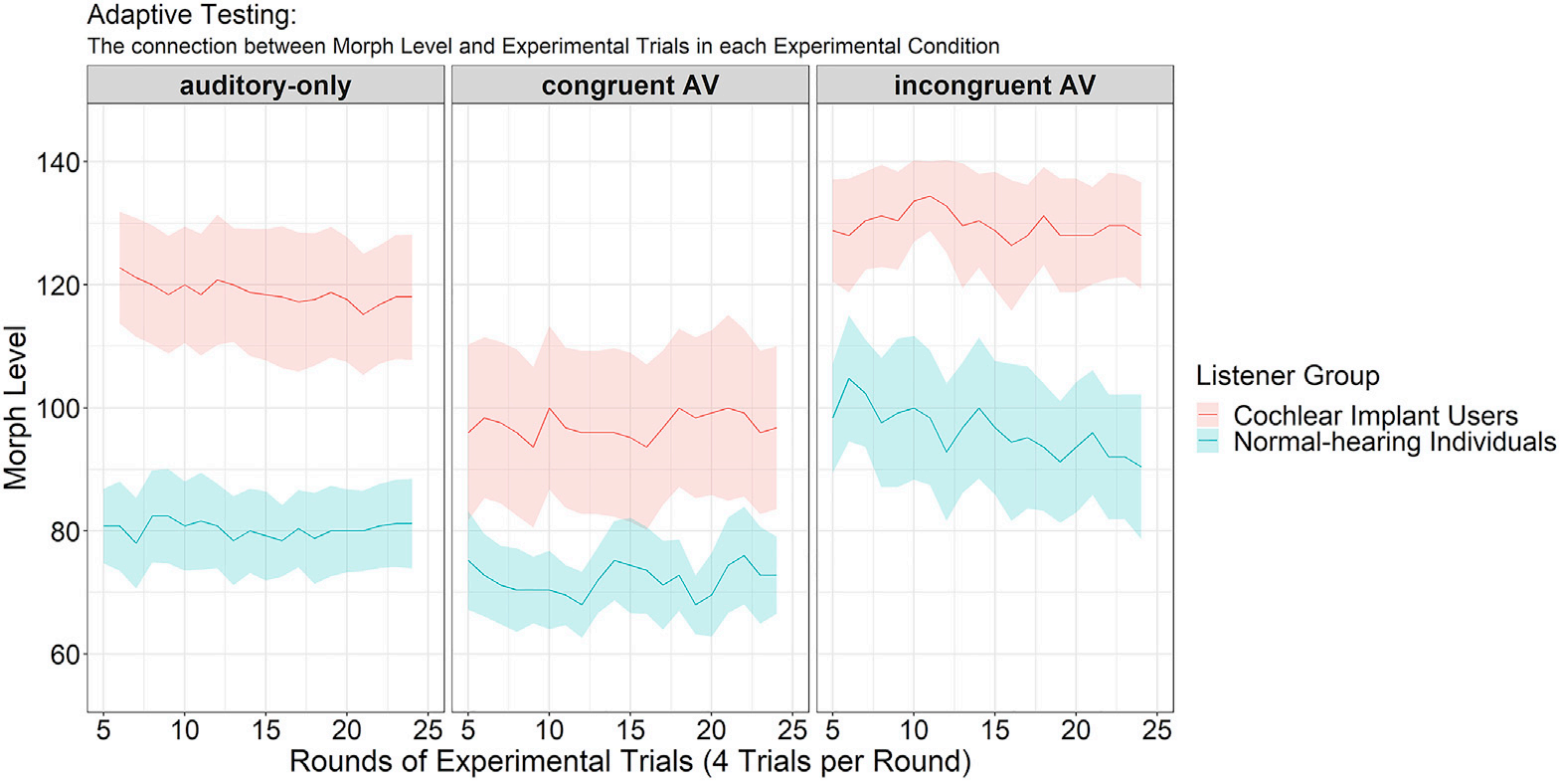
Experiment 2. Mean morph levels achieved by CI users (in red) and NH individuals (in turquoise) in the adaptive testing procedure which was targeted at 75% correct responses across consecutive rounds of experimental trials (N = 4 each) Note that lower morph levels represent better performance. Note substantial benefits from congruent AV information in CI users. Colored areas represent the 95% confidence intervals.

CI users compared to NH individuals performed on higher MLs (i.e., needed more diagnostic vocal information for recognizing 75% of items correctly) in all conditions (for auditory-only, AV congruent, and AV incongruent, *M*_CI_ ± SEM (standard error of the mean) = 118.9 ± 4.50, 97.08 ± 5.67, and 129.76 ± 3.59 and *M*_NH_ ± SEM = 80.30 ± 2.72, 72.04 ± 1.86, and 96.28 ± 4.27, respectively. This was reflected in a main effect LGroup: *F*(1, 48) = 45.294, p < 0.001, η_*p*_^*2*^ = 0.485; |*t*s(48)| ≥ 6.006, *p*s < 0.001, for auditory-only and AV incongruent stimuli; *t*(29.10) = 4.198, p < 0.001, for AV congruent stimuli). As Figure 2 suggests, benefits from visual congruent facial information appeared to be larger in CI users than in NH individuals (two-way interaction LGroup x Condition, *F*(2, 96) = 3.791, p = 0.042, ε_*HF*_ = 0.703, η_*p*_^*2*^ = 0.073).

#### CI users show strong benefits to vocal emotion perception if congruent facial information is available

We quantified benefits and costs from AV congruent and incongruent facial information, respectively, by calculating differences between MLs for congruent AV minus auditory-only and for incongruent AV minus auditory-only conditions (for each experimental trial round and participant). CI users gained more AV benefit (AV minus auditory-only) from congruent faces than NH individuals; *t*(38.501) = −2.909, p = 0.003. No prominent AV costs were observed for incongruent stimuli; *t*(48) = −1.481, p = 0.927.

#### CI users benefit more from congruent facial information even when auditory-only performance is equal to NH individuals

Note that not all participants approached MLs in each condition at which 75% correct responses were maintained. This was because some CI users underperformed even at the highest ML of 140%, and also because some NH individuals overperformed even at the lowest ML of 60%. Accordingly, we created subgroups of CI users and NH individuals with equal auditory-only performance levels, by iteratively removing pairs of the lowest-performing CI user and the highest-performing NH user, until the mean difference in ML between the groups was minimal. At this point, the difference in auditory-only performance was virtually eliminated (*M*_CI_ = 94.4 ± 4.61 vs. *M*_NH_ = 91.8 ± 4.79; *t*(18) = 0.391, p = 0.700; n = 10 per subgroup). Crucially, CI users of this subgroup showed better AV congruent performance than did NH individuals; *M*_CI_ = 71.2 ± 1.79 vs. *M*_NH_ = 78.3 ± 3.50; *t*(18) = −1.808, p = 0.044. By contrast, CI users did not show lower AV incongruent performance; *M*_CI_ = 115.6 ± 6.93 vs. *M*_NH_ = 113.8 ± 7.09; *t*(18) = 0.182, p = 0.429.

#### Caricaturing improves CI users’ and NH individuals’ ability to recognize vocal emotions

To quantify how much each participant’s VER accuracy improved with increasing MLs, we computed an additional performance score, by calculating for each condition and participant, the difference in accuracy, *M*_Acc_, between the highest and the lowest ML the participant performed on and by dividing the resulting difference by the number of ML steps involved. The results attest to the efficiency of vocal caricaturing for VER with a CI: CI users gained significant benefit with increasing ML; *t*(24) = 4.144, p < 0.001, *t*(24) = 5.643, p < 0.001, and (Note that in the AV incongruent condition, data were only available for 14 CI users. This was because this condition was too difficult for 11 CI users to achieve >75% correct in a 4-trial round even at ML 140, such that data for lower MLs were unavailable.) *t*(13) = 3.708, p = 0.001 for auditory-only, AV congruent, and AV incongruent stimuli; *M*_CI_ ± SEM = 0.061 ± 0.015, 0.079 ± 0.014, and 0.135 ± 0.036, respectively. As expected, VER accuracy also increased with ML for NH individuals; |*t*s(24)| ≥ 8.352, *p*s < 0.001, for auditory-only, AV congruent, and AV incongruent; *M*_NH_ ± SEM = 0.146 ± 0.010, 0.119 ± 0.011, and 0.136 ± 0.016, respectively. For full details, see the associated OSF Repository (OSF: https://osf.io/75wxq/).

#### CI users’ ability to perceive vocal emotions from lower MLs tends to be correlated with the quality of life domain environmental health

In CI users, there was a marginal negative correlation between the MLs in the auditory-only condition and the environmental health domain of the WHOQOL-BREF; *r*_*s*_ = −0.38, p = 0.063, n = 24. Note that because lower MLs reflect better performance, a negative correlation reflects a positive relationship between VER and QoL.

## Discussion

As an influential recent review on communication with a CI puts it, “the role of voice emotion perception and production in communication cannot be overstated”,^18^ (p. 37). The present research corroborates substantially lower VER skills in adult CI users as a group, compared to NH individuals, in line with earlier findings^34^^,^^39^^,^^40^; see Jiam et al.^18^ for a review. At the same time, we observed large interindividual differences in VER skills and confirmed that emotion recognition performance is positively related to quality of life.^19^^,^^20^

In Experiment 1—in which all participants were exposed to vocal emotional stimuli with unmanipulated intensities—we also found a substantially larger crossmodal benefit to VER from emotion-congruent facial videos in CI users than in NH controls. A crossmodal cost to VER induced by emotion-incongruent facial videos was also observed, but this effect was only marginally larger in CI users. At a general level, our findings in Experiment 1 appear to support the notion that crossmodal plasticity allowed for efficient AV integration, in tune with a number of other studies on the perception of speech^22^^,^^24^^,^^25^ or speaker gender.^26^ More specifically, however, the results of Experiment 1 could be seen in potential discrepancy with those by Fengler et al.,^32^ who observed rather consistent crossmodal costs from emotion-incongruent facial videos but less consistent benefits from emotion-congruent facial videos in CI users. These different results might be related to differences in the dependent variables (i.e., proportion correct vs. inverse efficiency scores) or other methodological factors; for instance, the conditions to observe crossmodal benefits in the present study could have been promoted by the precise temporal synchronization.^41^^,^^42^ Nevertheless, Experiment 1 cannot fully exclude the possibility that larger AV benefits in CI users could simply have resulted from their low baseline levels of auditory-only performance, following the well-known principle of *inverse effectiveness* in multisensory integration,^43^ (but see also Ross et al.^35^). According to this principle, weak auditory representations would be bound to benefit strongly from comparatively salient visual cues.

With the present Experiment 2, we can exclude this possibility that larger AV benefits in CI users simply reflect the principle of inverse effectiveness. Importantly, we used adaptive testing in combination with voice morphing as a method to equate auditory-only performance between CI users and NH listeners, which avoids the necessity to acoustically degrade stimuli via vocoding or adding of noise. In this situation, we found no convincing evidence to suggest that CI users exhibit larger costs from emotion-incongruent facial information than do NH listeners. Remarkably, even in this situation, we do find that CI users show an enhanced *crossmodal benefit* to VER induced by emotion-congruent facial videos. These findings provide strong support for the suggestion that crossmodal processing offers adaptive benefits to speech perception with a CI (as discussed above) and extends this notion into the domain of vocal emotion perception.

The present findings advocate AV stimuli to enhance and train both speech comprehension and socio-emotional communication with a CI, and we propose this should be explored further particularly for children with a CI.^44^ The present study also provides an intriguing perspective of using auditory morphing to improve vocal emotion recognition. While a recent study^38^ showed that digital caricaturing of vocal emotions linearly enhances VER in NH individuals, Experiment 2 provides important evidence to show that CI users' VER performance can benefit from vocal caricaturing. In the domain of face perception, we note that digital caricaturing is now considered as a general method to improve poor face recognition.^45^ Of relevance to the present study, facial caricaturing technology has also been applied successfully to enhance face recognition in the context of sensory loss such as in age-related macular degeneration.^46^ In audition, the idea to use digitally modified stimuli with enhanced diagnostic information to improve communication in individuals with sensory or central handicaps goes back at least to influential work by Tallal et al.,^47^ who targeted speech comprehension in language-learning impaired (LLI) children. These authors demonstrated large training benefits—corresponding to approximately 2 years of developmental age—to speech comprehension following daily training over only four weeks with temporally modified speech. In face perception, a recent study also yielded promising results from caricature training in individuals with below-average skills in face recognition.^48^ Although future research is needed to fully explore the potential of auditory caricature training to improve VER, we see voice morphing as a promising general method to devise training programs for CI users, and we currently explore this possibility in more detail. Ultimately, as technology for real-time voice synthesis is constantly improving,^49^^,^^50^ this research may also contribute to refining CI sound processors to optimize socio-emotional communication.

### Limitations of the studies

Although the present findings have important potential implications, one limitation of these studies is that they do not include a direct cortical measure which could further specify the neuronal mechanisms of crossmodal benefits to VER in CI users. We anticipate that this will be an area of future investigations. Moreover, note that in the interest of an analysis of individual differences, we did not counterbalance response keys to emotion categories in our 2-AFC task between participants. As the present experiments did not include a visual-only condition, one concern could be that CI users would be better at emotion recognition from facial information and that this could have affected the present results. However, this seems unlikely: as discussed above, Fengler et al. (2017) showed that adult CI users did not outperform NH listeners in the recognition of dynamic facial emotional expressions. In fact, another recent study showed a reduction of emotional sensitivity to visual facial expressions in adult CI users.^51^ This corresponds to our own findings in pilot experimentation on a superset of the present stimuli, in which we had included a visual-only control condition (see Figure S1). Based on Experiment 1, it could be argued that AV benefits in NH listeners could have been limited because their performance may have been close to ceiling levels. However, we can exclude this in Experiment 2 in which performance was not at ceiling in NH listeners (for individual data, cf. Figure S2). Finally, it should be noted that VER performance in adult CI users is subject to large individual differences which likely have multiple origins and which this study cannot fully resolve. Among other factors, the specific CI hardware, the sound processor type, age at implantation, or duration of preimplantation deafness could all affect the results in the present experiments. Our visual inspection of the data did not suggest a clear pattern of relationships between any of these variables and experimental outcome. However, note that although the sample size of CI users in this study is substantial relative to other published studies in the field, it is not remotely sufficient to fully address the potential influence of such variables—thus calling for larger and ideally multicentric studies which currently are lacking in the field.

## STAR★Methods

### Key resources table

**Table:** 

REAGENT or RESOURCE	SOURCE	IDENTIFIER
**Deposited data**		

Raw data (deposited in the associated OSF Repository, https://osf.io/75wxq/)	this manuscript	https://osf.io/75wxq/
Analysis scripts (deposited in the associated OSF Repository, https://osf.io/75wxq/)	this manuscript	https://osf.io/75wxq/

**Software and algorithms**		

R	R Core Team, 2020^52^	https://www.r-project.org/
G∗Power 3.1	Faul et al., 2009^53^	https://www.psychologie.hhu.de/arbeitsgruppen/allgemeine-psychologie-und-arbeitspsychologie/gpower
digital audio test	Cotral-Labor-GmbH, 2013^54^	www.cotral.com/Hoertest/Hoertest.exe
STRAIGHT morphing technology	Kawahara et al., 2013;^36^ Kawahara and Skuk, 2019^37^	https://github.com/HidekiKawahara/legacy_STRAIGHT
E-Prime® 3.0	https://pstnet.com/products/e-prime/	https://pstnet.com/products/e-prime/

**Other**		

Stimulus items	this manuscript	https://osf.io/75wxq/

### Resource availability

#### Lead contact

Further information and requests for resources and reagents should be directed to and will be fulfilled by the lead contact, Celina I. von Eiff (celina.isabelle.von.eiff@uni-jena.de).

#### Materials availability

In the main paper, we report results which were of primary interest for the study purpose. Extensive further supplemental information (e.g., examples of stimuli, figures, scripts, raw data) can be found in the associated OSF Repository (https://osf.io/75wxq/).

### Experimental model and subject details

The studies were approved by the Ethics Committee of Jena University Hospital (Reference Number 2019-1606_1-BO). At the beginning of the experiments, all participants gave written informed consent after being carefully informed about the procedure and the aim of the studies, that all data were rendered pseudonymized, that results of the studies might be published in a scientific journal, and that participation was voluntary and could be discontinued at any time if they wished so.

#### Experiment 1

##### Participants

We planned the study to have sufficient statistical power to detect a medium-sized (*f* = 0.25) interaction between group (2) and condition (3) at an alpha level of 0.05 with a power of at least 0.80, using G∗Power 3.1.^53^ This resulted in a minimum required sample size of n = 14 per group. We tested 26 (17 female) CI users aged between 20 and 82 years (*M* = 55.65, *SD* = 16.12) and 26 (17 female) individuals with NH abilities aged between 20 and 82 years (*M* = 55.50, *SD* = 16.33), closely matched to CI users for age and gender. One group of participants was tested at the Cochlear Implant Rehabilitation Center Thuringia in Erfurt, another at Jena University Hospital. The latter received a small financial reimbursement to compensate for local travel expenses. All participants were native German speakers without neurological or psychiatric diagnoses. CI users reported no other otologic disorders and had either bilateral implants or unilateral implants and a severe to profound (>71 dB HL) hearing loss in the non-implanted ear. A digital audio test^54^ was used to confirm absence of hearing loss in controls.

#### Experiment 2

##### Participants

Using analogous considerations regarding statistical power and sample planning as in Experiment 1, we tested twenty-five (15 female) CI users aged between 25 and 70 years (*M* = 50.36, *SD* = 13.30) and 25 (15 female) NH controls aged between 26 and 70 years (*M* = 50.32, *SD* = 13.80), closely matched to CI users for age and gender. One group of participants was tested in the Cochlear Implant Rehabilitation Center Thuringia in Erfurt, another at Jena University Hospital. The latter received a small financial reimbursement to compensate for local travel expenses. Inclusion criteria were the same as in Experiment 1.

### Method details

#### Experiment 1

##### Stimuli

We selected all stimuli from a database we had created using emotion induction (rather than posed expressions), with high-quality video and audio recordings of 12 speakers (6 female) speaking 4 different phonetically balanced pseudowords (/belam/,/namil/,/molen/,/loman/) with 6 naturalistic basic emotions (anger, fear, happiness, disgust, sadness, surprise) plus a neutral emotion. The subset of emotions (anger, surprise) and speakers (8 speakers, 4 female) used in this study was chosen based on classification rates in a pilot study in which 4 CI users and 22 NH individuals rated the stimuli in an auditory-only, a visual-only, and a congruent AV condition. We used STRAIGHT morphing technology^36^^,^^37^ – which generates highly naturally sounding synthesized voices – to precisely time-synchronize the audio files with the videos in AV stimuli of congruent or incongruent expressions (for details on the synchronization procedure via temporal morphing, see supplemental material, 2.2). To ensure equal length of voice and dynamic face information, approximately 600 ms before voice onset and 800 ms after voice offset consisted of a silent blurred video. We presented 4 stimuli conditions: AV congruent (containing faces expressing the same emotion as the voices), AV incongruent (containing faces expressing the different emotion as the voices), auditory-only with original timing (i.e., timing of the faces expressing the same emotion as the voices), and auditory-only with “incongruent” timing (i.e., timing of the faces expressing the different emotion as the voices). Thus, all voices which were presented in AV conditions were also presented in the auditory-only conditions. Altogether, 256 stimuli (2 emotions x 8 speakers x 4 pseudowords x 4 conditions) were presented in the experiment. Mean duration of the stimuli was 2133 ms (*SD* = 118 ms, range: 1921 to 2593 ms).

##### Experimental setting

All participants performed the experiment using the same technical equipment, including a Fujitsu LIFEBOOK E754 notebook with a 32-bit operating system, an Intel Core i5-4210M CPU processor (2.60 GHz), 1.600 MHz, 500 GB/8 GB SSD-Cache, and a 39.6 cm (15.6″) HD display. Voice stimuli were presented binaurally in mono at a peak intensity of approximately 70 dB(A) SPL, as measured with a Brüel and Kjær Precision Sound Level Meter Type 2206, using two Creative Inspire T10 loudspeakers (5 Watts RMS per channel, 2 channels, 80 Hz–20 kHz). Subjects were tested individually in a sound-attenuated chamber (∼4 m^2^), with 1 m between head and monitor, with loudspeakers placed next to both monitor sides.

##### Procedure

Experimental sessions lasted approximately 35 min for CI users and 25 min for NH individuals, with the computer experiment lasting about 20 min. All participants filled in a self-report questionnaire on demographic data. CI users additionally answered questions regarding their personal experience with their CIs and filled in the 26-item WHOQOL-BREF questionnaire^55^ to assess quality of life. We then presented a computer experiment programmed with E-Prime® 3.0. For its duration, unilateral CI users were asked to turn off any hearing aids in the contralateral ear to avoid the contribution of residual hearing. CI users used the same CI sound processor(s) as in their daily routines. They performed a two-alternative forced choice (2-AFC) task, discriminating between surprise and anger. Experimental instructions were delivered via a monitor before the experiment. We asked participants to focus carefully on each stimulus and to decide as accurately and fast as possible whether it sounded angry or surprised. We emphasized that participants should always attend to the emotion in the *voice* and ignore the emotion expressed in the face in AV conditions as it was task-irrelevant. We emphasized, however, that they should always look at the faces. To discourage strategies to reduce visual input (e.g., closing eyes, looking away), the experimenter supervised the experiment in the same room. Pressing the corresponding keys "F" and "K" (German layout) indicated that the participant perceived the voice as surprised or angry, respectively. We used no counterbalancing of key assignments to emotion categories, to avoid confounds of individual differences with differences in experimental procedures. Sixteen practice trials with feedback about the accuracy of the previous response were presented to ensure that instructions were fully understood. After the experimenter reassured that the participant did not have remaining questions, experimental trials (in which no feedback on accuracy was given) were presented in 4 blocks of 64 trials each. Self-paced breaks were allowed after each block. All stimuli were presented once in random order. Each trial started with a green fixation cross which was replaced by either an AV stimulus or a green question mark after 500 ms. The onset of the question mark coincided with the onset of an auditory-only stimulus and remained on screen until the offset of the auditory-only stimulus.

#### Experiment 2

##### Stimuli

We selected half of the stimuli which were presented in Experiment 1 by choosing two pseudowords (/belam/,/molen/). Additionally, systematically altering all acoustic parameters (i.e., F0, formant frequencies, spectrum level, aperiodicity, and time) on the morph trajectory intersecting anger and surprise in all selected stimuli with TANDEM-STRAIGHT,^36^^,^^37^ we created synchronized AV stimuli with graded congruence of AV expressions. Accordingly, we varied the degree of “diagnostic” emotional information in a voice in 20% steps from emotion caricatures over original voices to emotion anti-caricatures (140%, 120%, 100%, 80%, or 60%), corresponding to decreasing emotion intensity. Thus, whereas we presented the same stimuli conditions as we did in Experiment 1 (i.e., AV congruent, AV incongruent, auditory-only with original timing, auditory-only with “incongruent” timing) in principle, in Experiment 2, all conditions contained stimuli with graded intensity. Altogether, the experiment contained 640 stimuli (2 emotions x 8 speakers x 2 pseudowords x 5 ML x 4 conditions). Mean duration of stimuli was 2130 ms (*SD* = 128 ms, range: 1921 to 2593 ms).

##### Experimental setting

Experimental setting was analogous to the one in Experiment 1.

##### Procedure

Experimental sessions lasted approximately 45 min for CI users and 35 min for NH individuals, with the computer experiment lasting about 30 min. The procedure was identical to Experiment 1, with the exception that we presented an experiment with a unique adaptive testing procedure to the participants. An algorithm selected subsequent stimuli based on each participant’s performance in the previous four trials (per condition), to asymptotically approach MLs in each condition at which 75% of items were correct. For each upcoming 4-trial series, the algorithm selected stimuli with more diagnostic emotional information for <75% (i.e., 2 or less) trials correct, stimuli with equivalent diagnostic emotional information for performance at 75% (i.e., 3) trials, and stimuli with less diagnostic emotional information for >75% (i.e., all 4) trials correct in each preceding 4-trial series. Altogether, 384 stimuli (96 trials/condition) were presented to each participant in the experiment, in 8 blocks of 48 trials each, with conditions presented in random order. Eight practice trials (with feedback on accuracy of the previous response).

### Quantification and statistical analysis

#### Experiment 1

##### Statistical analysis

Statistical analyses were performed using R.^52^ Trials with reaction times >6000 ms (from voice offset; 0.87% of experimental trials) were excluded. Where appropriate, we performed Epsilon corrections for heterogeneity of covariances.^56^ Due to the robustness of ANOVAs to violations of normality,^57^ we did not test for distribution assumptions otherwise. In case of significant two-way interactions, we conducted post-hoc analyses by using two-tailed *t* tests or, where appropriate, Welch tests (comparing CI users and NH individuals or experimental conditions). We used one-tailed *t* tests for comparisons we predicted according to the study's preregistration at OSF (https://osf.io/dpwbq). As recognition accuracy did not differ significantly between the two auditory-only conditions for the overall sample (*t*(51) = −0.998, p = 0.323), or separately for CI users or NH individuals only, |*t*s(25)| ≤ 1.069, *p*s ≥ 0.295, we combined them to one auditory-only condition by calculating the mean accuracy across the two conditions (which only differed in their timing). Moreover, we collapsed data across listener sex (LSex: female, male) because an initial overall ANOVA on recognition accuracy did not indicate any main effects or interactions involving LSex (all *p*s ≥ 0.368). Thus, the final mixed ANOVA included the within-subject factors modality (auditory-only, AV congruent, AV incongruent), speaker sex (SpSex: female, male), and the between-subject factor listener group (LGroup: CI, NH).

#### Experiment 2

##### Statistical analysis

Statistical analysis was analogous to Experiment 1. A proportion of 0.26% of experimental trials were excluded from analyses due to reaction times >6000 ms (from voice offset). As the two auditory-only conditions did not significantly differ in MLs for all participants taken together, *t*(49) = −1.710, p = 0.094, or separately for CI users or NH individuals only, |*t*s(24)| ≤ 1.707, *p*s ≥ 0.101, we combined them to one auditory-only condition by calculating mean MLs (across the two MLs in an experimental trial round). The two auditory-only conditions did also not significantly differ in recognition accuracy for all participants taken together, *t*(49) = −0.199, p = 0.843, or separately for CI users or NH individuals only, |*t*s(24)| ≤ 0.812, *p*s ≥ 0.425, so that we additionally combined them to one auditory-only condition by calculating mean accuracy on each ML, to later conduct analyses with recognition accuracy as dependent variable. As we aimed at only analysing experimental rounds in which participants had approximately reached their ML in the adaptive testing procedure, we excluded data from the first four rounds per condition. We moreover collapsed data across listener sex (LSex: female, male) for analyses because the initial 3 × 2 × 2 mixed ANOVA on ML, with within-subject factor experimental condition (Condition: auditory-only, AV congruent, AV incongruent), and between-subject factors listener sex and listener group (LGroup: CI, NH) did not reveal any main effects or interactions involving LSex (all *p*s ≥ 0.118). We used one-tailed *t* tests for pairwise comparisons we predicted according to the study's preregistration at OSF (https://osf.io/brnp8).

## Data Availability

• Raw data have been deposited in the associated OSF Repository (OSF: https://osf.io/75wxq/) and are publicly available as of the date of publication. • All scripts have been deposited at OSF: https://osf.io/75wxq/ and are publicly available as of the date of publication. • Any additional information required to reanalyze the data reported in this paper is available from the lead contact upon request.
