# Supplementary material for: Crossmodal benefits to vocal emotion perception in cochlear implant users
Source: iScience. 2022 Dec 2;25(12):105711. doi: 10.1016/j.isci.2022.105711 (PMC9791346; doi:10.1016/j.isci.2022.105711)
Supplement: Document S1. Figures S1 and S2 and Tables S1 and S2 [file mmc1.pdf]

iScience, Volume *25*

Supplemental information

Crossmodal benefits to vocal emotion perception in cochlear implant users

Celina Isabelle von Eiff, Sascha Frühholz, Daniela Korth, Orlando Guntinas-Lichius, and Stefan Robert Schweinberger

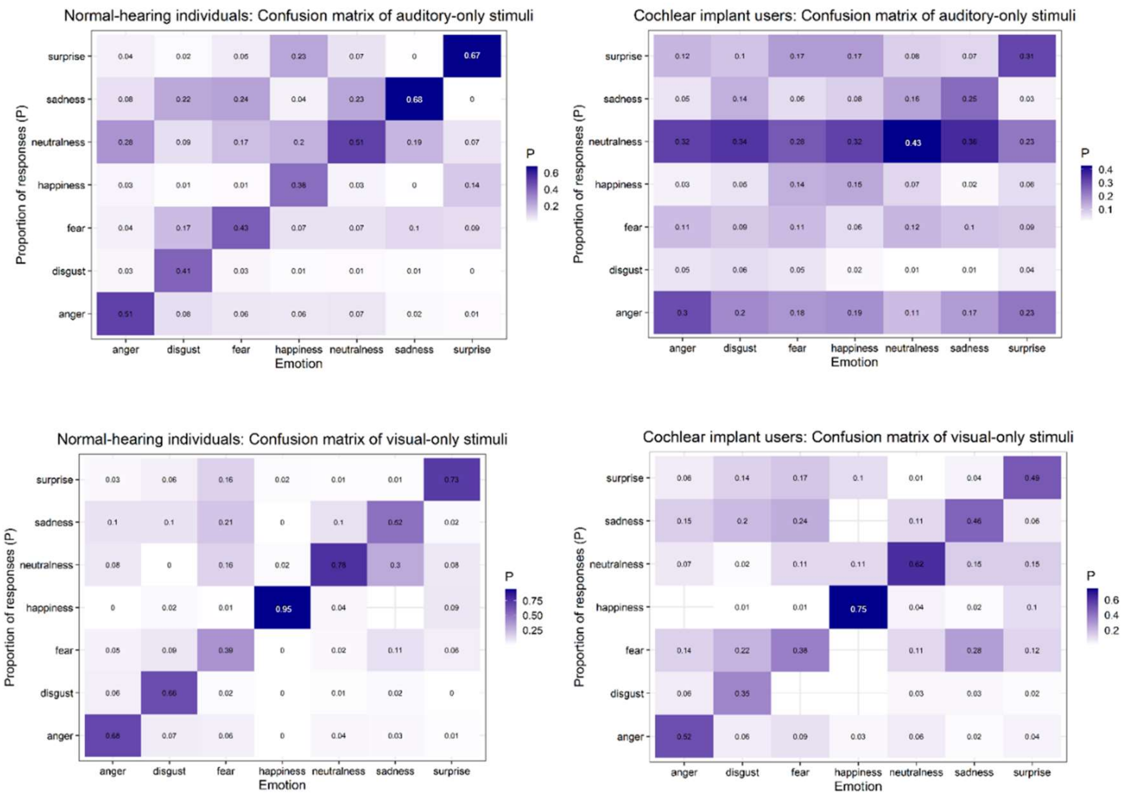

Figure S1. Classification performance of CI users (N = 4) and normal-hearing individuals (N = 22) for auditory-only and visual-only stimuli of the rating study, Related to Limitations of the studies and STAR Methods

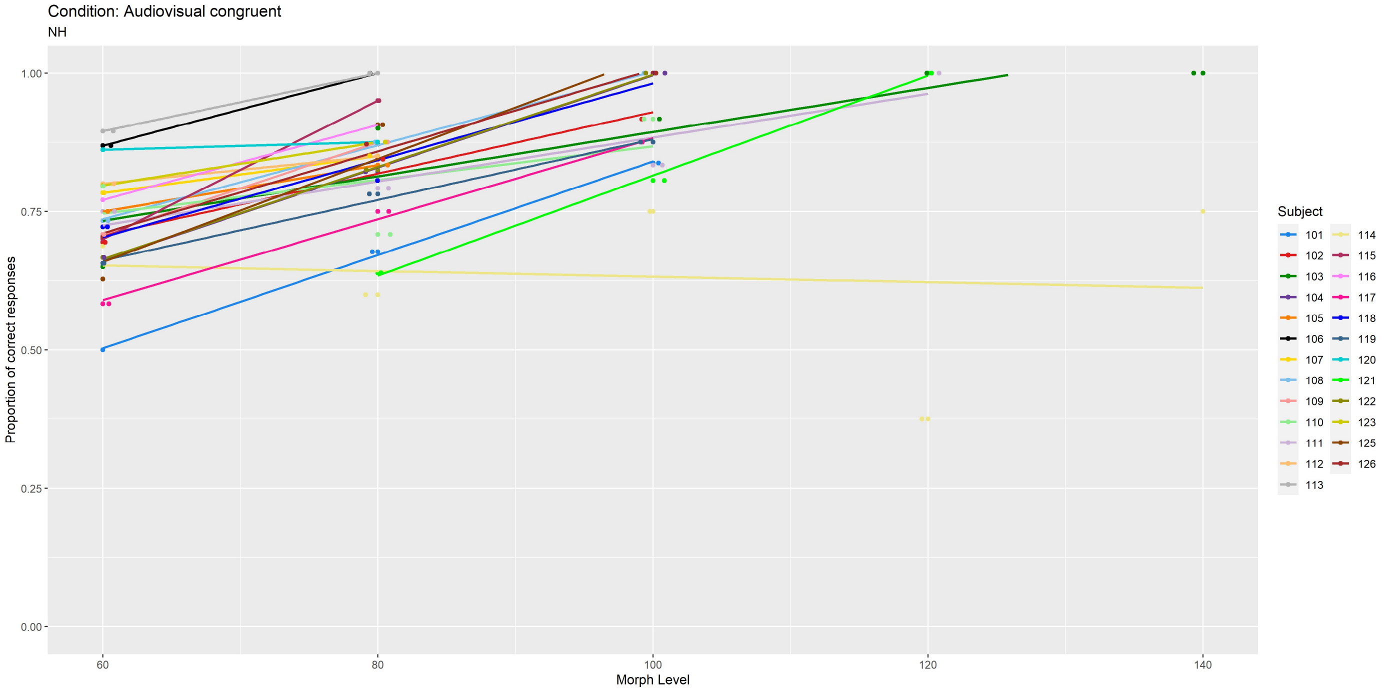

Figure S2. Experiment 2. Proportion of correct responses achieved at each morph level in Experiment 2, audiovisual congruent condition. Colors denote individual normal-hearing (NH) individuals., Related to Limitations of the studies and STAR Methods

Table S1. Time anchor points of auditory stimuli, Related to STAR Methods

Pseudoword	Speaker	Vocal and Facial Emotion	TA 1	TA 2	TA 3	TA 4	TA 5	TA 6	TA 7	TA 8	TA 9	TA 10	TA 11	TA 12
belam	WK01	VA FA (cong)	0.51	0.526	0.592	0.664	0.71	0.753	0.793	0.83	0.866	0.903	1.018	1.149
		VS FA (incong)	0.51	0.526	0.592	0.664	0.71	0.753	0.793	0.83	0.866	0.903	1.018	1.149
		VS FS (cong)	0.545	0.554	0.61	0.664	0.698	0.732	0.77	0.805	0.841	0.88	0.924	0.97
		VA FS (incong)	0.545	0.554	0.61	0.664	0.698	0.732	0.77	0.805	0.841	0.88	0.924	0.97
belam	HO05	VA FA (cong)	0.543	0.562	0.613	0.663	0.703	0.743	0.808	0.876	0.943	1.009	1.089	1.167
		VS FA (incong)	0.543	0.562	0.613	0.663	0.703	0.743	0.808	0.876	0.943	1.009	1.089	1.167
		VS FS (cong)	0.521	0.587	0.636	0.686	0.732	0.775	0.839	0.899	0.962	1.024	1.11	1.196
		VA FS (incong)	0.521	0.587	0.636	0.686	0.732	0.775	0.839	0.899	0.962	1.024	1.11	1.196
belam	MK06	VA FA (cong)	0.533	0.548	0.607	0.67	0.715	0.755	0.811	0.871	0.928	0.988	1.053	1.121
		VS FA (incong)	0.533	0.551	0.635	0.72	0.768	0.811	0.872	0.933	0.992	1.052	1.145	1.234
		VS FS (cong)	0.533	0.551	0.635	0.72	0.768	0.811	0.872	0.933	0.992	1.052	1.145	1.234
		VA FS (incong)	0.533	0.548	0.607	0.67	0.715	0.755	0.811	0.871	0.928	0.988	1.053	1.121
belam	JW07	VA FA (cong)	0.516	0.54	0.649	0.763	0.796	0.831	0.906	0.983	1.057	1.129	1.196	1.26
		VS FA (incong)	0.516	0.54	0.649	0.763	0.796	0.831	0.906	0.983	1.057	1.129	1.196	1.26
		VS FS (cong)	0.528	0.552	0.6	0.653	0.684	0.713	0.746	0.782	0.819	0.854	0.942	1.028
		VA FS (incong)	0.528	0.552	0.6	0.653	0.684	0.713	0.746	0.782	0.819	0.854	0.942	1.028
belam	LN08	VA FA (cong)	0.509	0.532	0.58	0.625	0.681	0.736	0.805	0.874	0.939	1.003	1.13	1.253
		VS FA (incong)	0.509	0.532	0.58	0.625	0.681	0.736	0.805	0.874	0.939	1.003	1.13	1.253
		VS FS (cong)	0.539	0.555	0.594	0.631	0.664	0.69	0.768	0.851	0.939	1.022	1.127	1.232
		VA FS (incong)	0.539	0.555	0.594	0.631	0.664	0.69	0.768	0.851	0.939	1.022	1.127	1.232
belam	MB10	VA FA (cong)	0.541	0.557	0.597	0.636	0.675	0.715	0.762	0.808	0.859	0.906	0.991	1.074
		VS FA (incong)	0.541	0.557	0.597	0.636	0.675	0.715	0.762	0.808	0.859	0.906	0.991	1.074
		VS FS (cong)	0.542	0.559	0.591	0.624	0.654	0.688	0.742	0.794	0.848	0.913	0.966	1.026
		VA FS (incong)	0.542	0.559	0.591	0.624	0.654	0.688	0.742	0.794	0.848	0.913	0.966	1.026
belam	LG11	VA FA (cong)	0.527	0.544	0.584	0.62	0.66	0.701	0.763	0.823	0.884	0.943	1.019	1.102
		VS FA (incong)	0.527	0.544	0.584	0.62	0.66	0.701	0.763	0.823	0.884	0.943	1.019	1.102
		VS FS (cong)	0.546	0.569	0.603	0.635	0.69	0.748	0.819	0.89	0.962	1.036	1.11	1.182
		VA FS (incong)	0.546	0.569	0.603	0.635	0.69	0.748	0.819	0.89	0.962	1.036	1.11	1.182
belam	TL12	VA FA (cong)	0.515	0.529	0.561	0.593	0.619	0.645	0.682	0.72	0.759	0.798	0.87	0.943
		VS FA (incong)	0.515	0.529	0.561	0.593	0.619	0.645	0.682	0.72	0.759	0.798	0.87	0.943
		VS FS (cong)	0.547	0.567	0.595	0.621	0.651	0.681	0.726	0.774	0.822	0.873	0.95	1.028
		VA FS (incong)	0.547	0.567	0.595	0.621	0.651	0.681	0.726	0.774	0.822	0.873	0.95	1.028
belam		*Mean*	*0.531*	*0.552*	*0.603*	*0.654*	*0.694*	*0.732*	*0.788*	*0.845*	*0.901*	*0.958*	*1.04*	*1.123*
molen	WK01	VA FA (cong)	0.526	0.62	0.7	0.771	0.861	0.95	1.012	1.075	1.143	1.21	1.364	1.523
		VS FA (incong)	0.526	0.62	0.7	0.771	0.861	0.95	1.012	1.075	1.143	1.21	1.364	1.523
		VS FS (cong)	0.53	0.635	0.702	0.769	0.829	0.901	0.957	1.012	1.068	1.126	1.214	1.301
		VA FS (incong)	0.53	0.635	0.702	0.769	0.829	0.901	0.957	1.012	1.068	1.126	1.214	1.301
molen	HO05	VA FA (cong)	0.539	0.618	0.69	0.761	0.797	0.833	0.884	0.935	0.992	1.048	1.112	1.177
		VS FA (incong)	0.539	0.618	0.69	0.761	0.797	0.833	0.884	0.935	0.992	1.048	1.112	1.177
		VS FS (cong)	0.539	0.576	0.626	0.677	0.706	0.737	0.792	0.854	0.914	0.976	1.073	1.163
		VA FS (incong)	0.539	0.576	0.626	0.677	0.706	0.737	0.792	0.854	0.914	0.976	1.073	1.163
molen	MK06	VA FA (cong)	0.539	0.587	0.669	0.751	0.803	0.851	0.906	0.96	1.015	1.072	1.157	1.246
		VS FA (incong)	0.539	0.587	0.669	0.751	0.803	0.851	0.906	0.96	1.015	1.072	1.157	1.246
		VS FS (cong)	0.532	0.623	0.691	0.762	0.826	0.893	0.946	0.995	1.053	1.11	1.16	1.212

		VA FS (incong)	0.532	0.623	0.691	0.762	0.826	0.893	0.946	0.995	1.053	1.11	1.16	1.212
molen	JW07	VA FA (cong)	0.534	0.617	0.681	0.745	0.78	0.816	0.878	0.942	1.003	1.067	1.133	1.206
		VS FA (incong)	0.534	0.617	0.681	0.745	0.78	0.816	0.878	0.942	1.003	1.067	1.133	1.206
		VS FS (cong)	0.529	0.62	0.666	0.713	0.744	0.774	0.836	0.901	0.971	1.041	1.108	1.175
		VA FS (incong)	0.529	0.62	0.666	0.713	0.744	0.774	0.836	0.901	0.971	1.041	1.108	1.175
molen	LN08	VA FA (cong)	0.525	0.654	0.693	0.735	0.823	0.914	0.97	1.025	1.082	1.138	1.242	1.341
		VS FA (incong)	0.525	0.654	0.693	0.735	0.823	0.914	0.97	1.025	1.082	1.138	1.242	1.341
		VS FS (cong)	0.52	0.586	0.612	0.639	0.678	0.722	0.769	0.819	0.873	0.923	1.015	1.108
		VA FS (incong)	0.52	0.586	0.612	0.639	0.678	0.722	0.769	0.819	0.873	0.923	1.015	1.108
molen	MB10	VA FA (cong)	0.552	0.615	0.652	0.686	0.722	0.758	0.804	0.851	0.898	0.948	1.02	1.098
		VS FA (incong)	0.552	0.615	0.652	0.686	0.722	0.758	0.804	0.851	0.898	0.948	1.02	1.098
		VS FS (cong)	0.525	0.59	0.643	0.702	0.73	0.755	0.798	0.846	0.894	0.943	1.021	1.096
		VA FS (incong)	0.525	0.59	0.643	0.702	0.73	0.755	0.798	0.846	0.894	0.943	1.021	1.096
molen	LG11	VA FA (cong)	0.542	0.605	0.65	0.696	0.743	0.791	0.841	0.892	0.94	0.991	1.067	1.139
		VS FA (incong)	0.542	0.605	0.65	0.696	0.743	0.791	0.841	0.892	0.94	0.991	1.067	1.139
		VS FS (cong)	0.536	0.628	0.681	0.735	0.789	0.846	0.901	0.957	1.013	1.067	1.136	1.214
		VA FS (incong)	0.536	0.628	0.681	0.735	0.789	0.846	0.901	0.957	1.013	1.067	1.136	1.214
molen	TL12	VA FA (cong)	0.516	0.563	0.598	0.634	0.66	0.686	0.729	0.773	0.823	0.871	0.928	0.985
		VS FA (incong)	0.516	0.563	0.598	0.634	0.66	0.686	0.729	0.773	0.823	0.871	0.928	0.985
		VS FS (cong)	0.523	0.621	0.66	0.699	0.731	0.763	0.808	0.857	0.901	0.949	1.034	1.126
		VA FS (incong)	0.523	0.621	0.66	0.699	0.731	0.763	0.808	0.857	0.901	0.949	1.034	1.126
molen		*Mean*	*0.532*	*0.610*	*0.663*	*0.717*	*0.764*	*0.812*	*0.864*	*0.918*	*0.974*	*1.03*	*1.112*	*1.194*
namil	WK01	VA FA (cong)	0.516	0.63	0.7	0.768	0.824	0.877	0.939	0.998	1.055	1.11	1.199	1.287
		VS FA (incong)	0.516	0.63	0.7	0.768	0.824	0.877	0.939	0.998	1.055	1.11	1.199	1.287
		VS FS (cong)	0.546	0.597	0.638	0.676	0.719	0.764	0.806	0.847	0.89	0.931	0.969	1.007
		VA FS (incong)	0.546	0.597	0.638	0.676	0.719	0.764	0.806	0.847	0.89	0.931	0.969	1.007
namil	HO05	VA FA (cong)	0.527	0.586	0.64	0.695	0.742	0.79	0.854	0.923	0.986	1.043	1.105	1.169
		VS FA (incong)	0.527	0.586	0.64	0.695	0.742	0.79	0.854	0.923	0.986	1.043	1.105	1.169
		VS FS (cong)	0.527	0.577	0.619	0.662	0.693	0.723	0.782	0.848	0.912	0.979	1.05	1.116
		VA FS (incong)	0.527	0.577	0.619	0.662	0.693	0.723	0.782	0.848	0.912	0.979	1.05	1.116
namil	MK06	VA FA (cong)	0.543	0.611	0.699	0.781	0.854	0.925	0.99	1.051	1.114	1.175	1.238	1.299
		VS FA (incong)	0.543	0.611	0.699	0.781	0.854	0.925	0.99	1.051	1.114	1.175	1.238	1.299
		VS FS (cong)	0.536	0.592	0.654	0.715	0.765	0.822	0.872	0.919	0.972	1.023	1.109	1.196
		VA FS (incong)	0.536	0.592	0.654	0.715	0.765	0.822	0.872	0.919	0.972	1.023	1.109	1.196
namil	JW07	VA FA (cong)	0.513	0.614	0.681	0.748	0.793	0.836	0.907	0.976	1.043	1.105	1.158	1.215
		VS FA (incong)	0.513	0.614	0.681	0.748	0.793	0.836	0.907	0.976	1.043	1.105	1.158	1.215
		VS FS (cong)	0.513	0.578	0.622	0.664	0.701	0.733	0.787	0.84	0.892	0.952	1	1.052
		VA FS (incong)	0.513	0.578	0.622	0.664	0.701	0.733	0.787	0.84	0.892	0.952	1	1.052
namil	LN08	VA FA (cong)	0.549	0.635	0.678	0.721	0.808	0.901	0.955	1.006	1.06	1.107	1.22	1.347
		VS FA (incong)	0.549	0.635	0.678	0.721	0.808	0.901	0.955	1.006	1.06	1.107	1.22	1.347
		VS FS (cong)	0.529	0.633	0.67	0.708	0.744	0.782	0.832	0.888	0.941	1.003	1.074	1.148
		VA FS (incong)	0.529	0.633	0.67	0.708	0.744	0.782	0.832	0.888	0.941	1.003	1.074	1.148
namil	MB10	VA FA (cong)	0.536	0.575	0.617	0.657	0.704	0.753	0.793	0.827	0.865	0.907	0.992	1.072
		VS FA (incong)	0.536	0.575	0.617	0.657	0.704	0.753	0.793	0.827	0.865	0.907	0.992	1.072
		VS FS (cong)	0.515	0.614	0.652	0.689	0.739	0.787	0.827	0.865	0.901	0.943	1.012	1.083
		VA FS (incong)	0.515	0.614	0.652	0.689	0.739	0.787	0.827	0.865	0.901	0.943	1.012	1.083
namil	LG11	VA FA (cong)	0.539	0.596	0.642	0.684	0.733	0.79	0.861	0.93	0.993	1.053	1.108	1.163
		VS FA (incong)	0.539	0.596	0.642	0.684	0.733	0.79	0.861	0.93	0.993	1.053	1.108	1.163

		VS FS (cong)	0.546	0.582	0.639	0.693	0.772	0.855	0.909	0.97	1.026	1.086	1.142	1.199
		VA FS (incong)	0.546	0.582	0.639	0.693	0.772	0.855	0.909	0.97	1.026	1.086	1.142	1.199
namil	TL12	VA FA (cong)	0.518	0.536	0.562	0.587	0.626	0.669	0.718	0.763	0.813	0.864	0.925	0.988
		VS FA (incong)	0.518	0.536	0.562	0.587	0.626	0.669	0.718	0.763	0.813	0.864	0.925	0.988
		VS FS (cong)	0.513	0.679	0.724	0.768	0.803	0.839	0.892	0.937	0.986	1.037	1.108	1.184
		VA FS (incong)	0.513	0.679	0.724	0.768	0.803	0.839	0.892	0.937	0.986	1.037	1.108	1.184
namil		*Mean*	*0.529*	*0.602*	*0.652*	*0.701*	*0.751*	*0.803*	*0.858*	*0.912*	*0.966*	*1.020*	*1.088*	*1.158*
loman	WK01	VA FA (cong)	0.542	0.714	0.814	0.922	0.98	1.04	1.128	1.216	1.291	1.375	1.417	1.455
		VS FA (incong)	0.542	0.714	0.814	0.922	0.98	1.04	1.128	1.216	1.291	1.375	1.417	1.455
		VS FS (cong)	0.545	0.577	0.616	0.656	0.697	0.738	0.782	0.831	0.88	0.93	0.993	1.055
		VA FS (incong)	0.545	0.577	0.616	0.656	0.697	0.738	0.782	0.831	0.88	0.93	0.993	1.055
loman	HO05	VA FA (cong)	0.545	0.578	0.627	0.677	0.735	0.793	0.851	0.911	0.97	1.032	1.104	1.183
		VS FA (incong)	0.545	0.578	0.627	0.677	0.735	0.793	0.851	0.911	0.97	1.032	1.104	1.183
		VS FS (cong)	0.514	0.552	0.588	0.627	0.669	0.708	0.768	0.829	0.89	0.952	1.034	1.113
		VA FS (incong)	0.514	0.552	0.588	0.627	0.669	0.708	0.768	0.829	0.89	0.952	1.034	1.113
loman	MK06	VA FA (cong)	0.529	0.617	0.715	0.816	0.864	0.913	0.965	1.02	1.073	1.131	1.194	1.261
		VS FA (incong)	0.529	0.617	0.715	0.816	0.864	0.913	0.965	1.02	1.073	1.131	1.194	1.261
		VS FS (cong)	0.517	0.577	0.648	0.719	0.77	0.824	0.884	0.949	1.01	1.081	1.132	1.185
		VA FS (incong)	0.517	0.577	0.648	0.719	0.77	0.824	0.884	0.949	1.01	1.081	1.132	1.185
loman	JW07	VA FA (cong)	0.515	0.6	0.65	0.705	0.761	0.817	0.888	0.965	1.038	1.112	1.173	1.24
		VS FA (incong)	0.515	0.6	0.65	0.705	0.761	0.817	0.888	0.965	1.038	1.112	1.173	1.24
		VS FS (cong)	0.52	0.586	0.621	0.651	0.697	0.747	0.815	0.883	0.947	1.013	1.059	1.101
		VA FS (incong)	0.52	0.586	0.621	0.651	0.697	0.747	0.815	0.883	0.947	1.013	1.059	1.101
loman	LN08	VA FA (cong)	0.515	0.623	0.664	0.703	0.767	0.829	0.898	0.964	1.029	1.101	1.21	1.327
		VS FA (incong)	0.515	0.623	0.664	0.703	0.767	0.829	0.898	0.964	1.029	1.101	1.21	1.327
		VS FS (cong)	0.516	0.609	0.638	0.667	0.695	0.717	0.779	0.846	0.911	0.98	1.083	1.189
		VA FS (incong)	0.516	0.609	0.638	0.667	0.695	0.717	0.779	0.846	0.911	0.98	1.083	1.189
loman	MB10	VA FA (cong)	0.534	0.577	0.603	0.629	0.673	0.716	0.766	0.813	0.864	0.916	0.982	1.052
		VS FA (incong)	0.534	0.577	0.603	0.629	0.673	0.716	0.766	0.813	0.864	0.916	0.982	1.052
		VS FS (cong)	0.502	0.583	0.617	0.654	0.69	0.728	0.773	0.818	0.869	0.923	1.007	1.091
		VA FS (incong)	0.502	0.583	0.617	0.654	0.69	0.728	0.773	0.818	0.869	0.923	1.007	1.091
loman	LG11	VA FA (cong)	0.528	0.646	0.691	0.732	0.79	0.856	0.918	0.984	1.049	1.115	1.189	1.263
		VS FA (incong)	0.528	0.646	0.691	0.732	0.79	0.856	0.918	0.984	1.049	1.115	1.189	1.263
		VS FS (cong)	0.53	0.604	0.66	0.716	0.77	0.826	0.894	0.964	1.036	1.112	1.192	1.273
		VA FS (incong)	0.53	0.604	0.66	0.716	0.77	0.826	0.894	0.964	1.036	1.112	1.192	1.273
loman	TL12	VA FA (cong)	0.511	0.541	0.561	0.583	0.614	0.65	0.704	0.763	0.817	0.876	0.921	0.965
		VS FA (incong)	0.511	0.541	0.561	0.583	0.614	0.65	0.704	0.763	0.817	0.876	0.921	0.965
		VS FS (cong)	0.512	0.624	0.652	0.678	0.712	0.752	0.798	0.848	0.899	0.951	1.028	1.109
		VA FS (incong)	0.512	0.624	0.652	0.678	0.712	0.752	0.798	0.848	0.899	0.951	1.028	1.109
loman		*Mean*	*0.523*	*0.601*	*0.648*	*0.696*	*0.743*	*0.791*	*0.851*	*0.93*	*0.973*	*1.038*	*1.107*	*1.179*

Note: We used twelve time anchor points of congruent vocal emotions to inform temporal morphing of incongruent vocal emotions towards the identical time structure. VA FA (cong) – “vocal anger, facial anger (congruent)”, VS FA (incong) – “vocal surprise, facial anger (incongruent)”, VS FS (cong) – “vocal surprise, facial surprise (congruent)”, VA FS (incong) – “vocal anger, facial surprise (incongruent)”, all values in seconds

Table S2. Demographic characteristics of CI users, Related to STAR Methods

								Left CI			Right CI		
CI user	Sex	Age	Civil status	Pre-/ post-deaf	Age at deafness	Age at first CI	Mode of hearing	Wear time (hr)	Manufacturer*	Processor	Wear time (hr)	Manufacturer*	Processor
							**Experiment 1**						
1	f	82	widowed	post	53	57	CI-bi	12-16	Cochlear	CP1000	12-16	Cochlear	CP910
2	f	78	widowed	pre	0	77	CI-right	/	/	/	8-12	AB	NaidaQ90
3	f	54	married	post	47	54	CI-right	/	/	/	> 16	MED-EL	Sonnet 2
4	f	73	married	post	51	73	CI-left	12-16	MED-EL	Sonnet 2	/	/	/
5	f	67	married	pre	0	30	CI-bi	> 16	MED-EL	Sonnet	> 16	MED-EL	OPUS2
6	f	69	married	post	57	69	CI-right	/	/	/	12-16	Cochlear	CP1000
7	f	60	widowed	post	50	59	CI-right	/	/	/	12-16	MED-EL	Rondo2
8	f	64	married	post	32	61	CI-bi	12-16	AB	NA	12-16	AB	NA
9	m	60	married	post	31	58	CI-left	12-16	MED-EL	Sonnet	/	/	/
10	m	61	single	pre	0	59	CI-right	/	/	/	8-12	Cochlear	CP1000
11	f	45	married	post	14	44	CI-right	/	/	/	8-12	MED-EL	Sonnet 2
12	f	60	married	post	59	60	CI-right	/	/	/	12-16	Cochlear	NA
13	m	65	married	post	20	64	CI-bi	12-16	Cochlear	CP1000	12-16	Cochlear	CP1000
14	m	34	single	post	20	31	CI-bi	> 16	MED-EL	Rondo	0-4	MED-EL	Sonnet
15	m	69	married	post	8	49	CI-bi	> 16	MED-EL	Sonnet	> 16	MED-EL	Sonnet
16	m	20	single	post	3	3	CI-left	> 16	MED-EL	Sonnet 2	/	/	/
17	f	51	single	post	6	46	CI-bi	> 16	AB	Q 70	> 16	AB	NaidaQ90
18	f	30	single	pre	0	26	CI-bi	12-16	Cochlear	CP910	12-16	Cochlear	CP1000
19	m	30	single	post	3	24	CI-bi	12-16	Cochlear	CP1000	12-16	Cochlear	CP1000
20	f	58	married	pre	2	39	CI-right	/	/	/	> 16	MED-EL	Sonnet
21	f	33	single	post	7	26	CI-left	12-16	Cochlear	CP1000	/	/	/
22	f	53	divorced	pre	0	48	CI-bi	> 16	MED-EL	Sonnet 2	> 16	MED-EL	Sonnet 2
23	f	70	married	post	49	49	CI-right	/	/	/	12-16	MED-EL	OPUS 2
24	m	67	married	post	43	55	CI-right	/	/	/	> 16	AB	NaidaQ90
25	m	54	divorced	pre	0	53	CI-left	> 16	Cochlear	CP1000	/	/	/
26	f	40	single	pre	0	38	CI-right	/	/	/	> 16	Cochlear	CP910
M		55.7			21.35	48.15							
SD		16.1			22.28	17.20							
Min		20			0	3							
Max		82			59	77							
N	26	26	26	26	26	26	26	26	26	26	26	26	26
							**Experiment 2**						
1	f	49	married	pre	0	44	CI-bi	> 16	Cochlear	CP1000	> 16	Cochlear	CP1000
2	f	62	married	post	48	61	CI-right	/	/	/	12-16	Cochlear	CP1000
3	m	58	married	post	30	50	CI-bi	> 16	Cochlear	CP1000	> 16	Cochlear	CP1000

4	f	40	single	pre	0	38	CI-right	/	/	/	> 16	Cochlear	CP910
5	m	43	single	post	19	25	CI-bi	12-16	MED-EL	NA	12-16	MED-EL	NA
6**	f	39	married	post	16	39	CI-left	> 16	MED-EL	Sonnet	/	/	/
7**	f	58	single	post	17	57	CI-left	> 16	AB	NaidaQ90	/	/	/
8**	f	45	single	post	41	45	CI-left	8-12	Cochlear	CP1000	/	/	/
9**	m	60	married	post	31	58	CI-left	12-16	MED-EL	Sonnet	/	/	/
10**	m	61	single	pre	0	59	CI-right	/	/	/	8-12	Cochlear	CP1000
11**	f	45	married	post	14	44	CI-right	/	/	/	8-12	MED-EL	Sonnet 2
12**	f	60	married	post	59	60	CI-right	/	/	/	12-16	Cochlear	NA
13	m	65	married	post	20	64	CI-bi	12-16	Cochlear	CP1000	12-16	Cochlear	CP1000
14	m	34	single	post	20	31	CI-bi	> 16	MED-EL	Rondo	0-4	MED-EL	Sonnet
15	m	69	married	post	8	49	CI-bi	> 16	MED-EL	Sonnet	> 16	MED-EL	Sonnet
16**	f	25	single	pre	0	2	CI-bi	> 16	MED-EL	Sonnet	> 16	MED-EL	Sonnet
17**	f	51	single	post	6	46	CI-bi	> 16	AB	Q 70	> 16	AB	NaidaQ90
18	f	30	single	pre	0	26	CI-bi	12-16	Cochlear	CP910	12-16	Cochlear	CP1000
19	m	30	single	post	3	24	CI-bi	12-16	Cochlear	CP1000	12-16	Cochlear	CP1000
20	f	58	married	pre	2	39	CI-right	/	/	/	> 16	MED-EL	Sonnet
21**	f	33	single	post	7	26	CI-left	12-16	Cochlear	CP1000	/	/	/
22	f	53	divorced	pre	0	48	CI-bi	> 16	MED-EL	Sonnet 2	> 16	MED-EL	Sonnet 2
23	f	70	married	post	49	49	CI-right	/	/	/	12-16	MED-EL	OPUS 2
24	m	67	married	post	43	55	CI-right	/	/	/	> 16	AB	NaidaQ90
25	m	54	divorced	pre	0	53	CI-left	> 16	Cochlear	CP1000	/	/	/
M		50.4			17.32	43.68							
SD		13.3			18.42	14.82							
Min		25			0	2							
Max		70			59	64							
N	25	25	25	25	25	25	25	25	25	25	25	25	25

CI, cochlear implant; CI-bi, bilateral implanted CI user; CI-left, unilateral implanted CI user who was fitted with the CI on the left ear; CI-right, unilateral implanted CI user who was fitted with the CI on the right ear; post = postlingually deafened; pre = prelingually deafened.

*CI Manufacturers: AB (Advanced Bionics GmbH), Max-Eyth-Str. 20, 70736 Fellbach-Oeffingen, Germany; Cochlear Headquarters, 1 University Avenue, Macquarie University, NSW, 2109, Australia; MED-EL Elektromedizinische Geräte Gesellschaft m.b.H., Fürstenweg 77a, 6020 Innsbruck, Austria.

** CI user was part of the subgroup.
